# PROTECTIVE EFFECTS OF NEIGHBORHOOD SOCIAL COHESION ON ELDER ABUSE IN INDIAN OLDER ADULTS

**DOI:** 10.1093/geroni/igz038.1580

**Published:** 2019-11-08

**Authors:** E-Shien Chang, Becca Levy

**Affiliations:** Yale School of Public Health, New Haven, Connecticut, United States

## Abstract

It is estimated that elder abuse impacts 16% of older persons globally. There is a need to understand factors that protect older persons. In this study, we examined whether neighborhood social cohesion, or the mutual support, trust, and interaction among neighbors, could be such a factor. As it has been found to be protective of child abuse and domestic violence, we hypothesized that higher neighborhood social cohesion would extend to reduce the risks of elder abuse. Our cohort consisted of participants aged 60 and over in the Longitudinal Aging Study in India (LASI) pilot survey. Elder abuse was measured by asking participants if they experienced ill-treatment by family members. Neighborhood social cohesion was measured by a five-item instrument that captured perceived support and trust among neighbors. The final sample consisted of 541 participants with a mean age of 69 who largely (72.9%) resided in rural area. The hypothesis was supported. Compared to older persons with low neighborhood social cohesion, older persons with high neighborhood social cohesion were significantly less likely to experience elder abuse (OR= 0.57, 95% CI=0.35-0.92), after controlling for socio-demographics, health, and neighborhood contextual covariates. This study, for the first time, suggests that neighborhood social environment may exert a protective effect on risks of elder abuse. Neighborhood resources may both prevent family members from being abusive and may help older persons stop abuse. Incorporating structural factors such as neighborhood cohesion may be critical in devising elder abuse preventions that increase the safety and well-being of older persons.

